# Boldness Personality Traits Are Associated With Reduced Risk Perceptions and Adoption of Protective Behaviors During the First COVID-19 Outbreak

**DOI:** 10.3389/fpsyg.2021.633555

**Published:** 2021-05-13

**Authors:** Tiago O. Paiva, Natália Cruz-Martins, Rita Pasion, Pedro R. Almeida, Fernando Barbosa

**Affiliations:** ^1^Laboratory of Neuropsychophysiology, Faculty of Psychology and Education Sciences, University of Porto, Porto, Portugal; ^2^Faculty of Law, Interdisciplinary Research Center on Crime, Justice and Security, School of Criminology, University of Porto, Porto, Portugal

**Keywords:** personality, psychopathic traits, COVID-19, risk perceptions, anxiety, protective behaviors

## Abstract

The containment measures imposed during the first COVID-19 outbreak required economic, social, and behavioral changes to minimize the spread of the coronavirus. Some studies have focused on how personality predicts distinct patterns of adherence to protective measures with psychopathic and antisocial traits predicting reduced engagement in such measures. In this study we extended previous findings by analyzing how boldness, meanness, and disinhibition psychopathic traits relate with both risk perceptions and protective behaviors during the first COVID-19 outbreak. A sample of 194 individuals (24% male) engaged in the survey, were assessed for psychopathic traits with the Triarchic Psychopathy Measure, and completed a COVID-19 survey targeting risk perceptions (spread, risk of becoming infected, state anxiety toward the COVID-19, and perceived risk of specific behaviors) and frequency of protective behaviors (e.g., not engaging in social distancing). Overall results show that boldness predicts reduced estimate of COVID-19 spread, reduced perceived risk of becoming infected, reduced state anxiety toward COVID-19, and reduced frequency of protective behaviors. Exploratory mediation models suggest that risk perceptions are not significant mediators of the association between psychopathic traits and reduced engagement in protective behaviors. Our results unveil that psychopathic traits affect risk perceptions and the propensity to engage in protective measures, emphasizing the need to accommodate these personality features in the public health strategy to control the COVID-19 spread.

## Introduction

Since its inception in December of 2019, the COVID-19 pandemic has left a devastating trail (Khalaf et al., 2020; Torequl Islam et al., 2020), with more than 94 million confirmed cases and more than 2 million deaths as of the second week of 2021 (ECDC, 2021). To slow down the spreading of the disease during the first outbreak, most countries responded to the COVID-19 spread by declaring state of emergency and mandatory confinement that markedly impacted the mental health of individuals (Mækelæ et al., 2020; Pfattheicher et al., 2020).

Despite the possible negative effects of confinement and social distancing, the governmental health control measures designed to prevent the spread of the virus play a pivotal role in controlling the COVID-19 pandemic. With notable few exceptions, over the last few months, public-health messages tried to increase the individual awareness and adherence to protective behaviors to which individuals reacted by diverse manners (Blagov, 2020). Being SARS-CoV-2 an invisible threat, individual behaviors may fall into one of two categories: (a) they may not consider it a real threat, and thus do not adhere to the protective control measures, such as ignoring social distancing, among others (Pfattheicher et al., 2020; Vieira et al., 2020; Wise et al., 2020); (b) individuals assume its ubiquity, and ultimately limit all their activities (Lanciano et al., 2020; Vieira et al., 2020). Both reactions have a considerable impact on both psychological functioning and behavior, ultimately triggering major issues on COVID-19 management and public health policies.

Being a predictor of health and health-related behaviors (Strickhouser et al., 2017), personality is one of the factors that may contribute to distinct individual reactions to government and public health-messages. In this context, psychopathic traits compose a set of personality features, including affective (e.g., lack of empathy, guilt, and deep emotional attachments to others) and interpersonal characteristics (e.g., superficial charm), as well as impulsive and antisocial behaviors (Hare and Neumann, 2008; Patrick and Drislane, 2015), that together have been linked to reduced risk perceptions and increased risk taking, including in health-related behaviors (Hudek-Knežević et al., 2007).

The modern operationalizations of psychopathy are inspired by the work of Cleckley, that in 1941 described several individuals who manifest severe impairments in personal and social functioning, but do not exhibit pathological externalizing symptoms, neither signs of dysfunctional reasoning (Cleckley, 1941). The characteristics of these individuals highlighted the presence of positive adjustment features, along with the display of persistent maladaptive behavior. Since then, neurobiological oriented models have provided evidence for two main etiological factors that underlie the expression of psychopathic traits: (a) dispositional fearlessness (or trait fearlessness), experimentally linked to a reduced reactivity to acute threats (Patrick, 1994; Blair, 2005; Paiva et al., 2020a); (b) externalizing vulnerability, experimentally linked to poor inhibitory control, and antisocial conducts (Patrick et al., 2009; Patrick and Bernat, 2010). Both dimensions represent two mechanisms that are critical for an adequate response to the COVID-19 challenges. On one hand, the trait fearlessness may boost the threat approach due to a reduced sensitivity to potentially harmful stimuli; on the other externalizing vulnerability entails lack of inhibitory control leading to risk taking and poor decision making. In this line, the triarchic operationalization of psychopathy defines three phenotypic constituents that characterize the psychopathic personality: (a) boldness, defined as the capacity to remain calm and focused under pressure or threat, ability to recover from stressful situations, high self-assurance, and social efficacy; (b) meanness, associated with the maladaptive interpersonal characteristics of psychopathy, including low empathy, weak attachments with others, rebelliousness, excitement seeking, exploitativeness, and empowerment through cruelty; and (c) disinhibition, defined as the tendency to display problems of impulse control, including lack of planfulness and foresight, impaired regulation of affect and urges, reliance on immediate gratification, and deficient behavioral restraint (Patrick et al., 2009). A recent study found that distinct triarchic psychopathic traits predict specific behavioral patterns in the context of the COVID-19 pandemic (Blagov, 2020). Specifically, while all traits predicted increased venturous behavior in a carrier scenario, only meanness and disinhibition have been associated with a reduced intent to engage in protecting others, also predicting lower predisposition to engage in social distancing and hygiene protective behaviors. In addition, the authors also reported that meanness and disinhibition were negatively correlated with the appeal of public health messages. An independent study assessing the impact of antisociality on behaviors during the COVID-19 outbreak reported that individuals with high antisocial levels have more risky behaviors, such as not adopting social distance measures, leaving home more often, and curiously approaching even more closely to people when in public areas, possibly denoting a lower level of concern of becoming infected or infecting other individuals (O’Connell et al., 2020). On a Brazilian sample, Miguel et al. (2021) also found that antisocial traits (callousness, deceitfulness, and risk-taking, and low empathy) were associated with less compliance with containment measures.

Overall, the putative mechanisms underlying the expression of psychopathic traits (cf. trait fearlessness and externalizing vulnerability) seem to potentiate risk-related behaviors and potentially reduce the adherence to public health measures. Indeed, the triarchic psychopathic traits have been linked to psychological mechanisms and relevant behaviors in the context of the COVID-19 pandemic, further supporting our hypotheses: (a) boldness is associated with reduced reported anxiety and threat sensitivity (Paiva et al., 2020b), possibly reducing risk perceptions and the frequency of protective behaviors (Pasion et al., 2020; H1); (b) meanness relates to reduced empathic concern toward others overall disregard for authority (Patrick et al., 2009; Paiva et al., 2020b), being possibly associated with lower perceived probability of infecting others and reduced protective behaviors (H2); and (c) disinhibition represents a close correlation to antisocial behavior (Patrick et al., 2009), possibly related to disregard for protective recommendations, and thus to a reduced frequency of protective behaviors (H3). Additionally, we intended to test the mediating role of risk perceptions on the associations between psychopathic traits and adoption of protective behaviors through exploratory mediation analysis. Thus, by reporting data on the personality determinants of perceptions and behaviors associated with the COVID-19 pandemic, the present study may inform better approaches to public outreach in controlling the present and future outbreaks.

## Materials and Methods

### Sample and Procedures

A total of 268 subjects enrolled in the study and were surveyed online. However, 69 participants did not complete any COVID-19-related section of the survey and were removed from the analysis. From the remaining 199 responses, we additionally excluded five participants that reported ages lower than 18 years old. The final sample included 194 individuals (24% male) with a mean age of 37.1 years old (*SD* = 14.5). Most of the sample completed the university (74.2%) or the secondary school (24.7%). For those actively working (71.6%) or studying (19.6%), 48.5% reported they were using online remote working. The zone of the residence covered the Portuguese national territory and was represented by both rural (27.8%) and urban areas (72.2%).

A power analysis estimate for linear multiple regression resulted on a sample size of 176 to detect effect sizes in the order of 0.3 (β = 0.95), suggesting that our sample size is adequate to detect effect sizes expected for correlations between self-report and behavioral measures (Hall et al., 2007).

Considering the recommendations for isolation and to minimize face-to-face interactions, participants were recruited by online advertisements on social media and on the university campus, and completed the survey online on Qualtrics (Qualtrics, Provo, UT, United States). The study was approved by the Local Ethics Committee and all participants gave informed consent before starting the survey.

### Survey Development and Measures

The survey questions were adapted from Pasion et al. (2020). The survey collected data on sociodemographic characteristics, perceptions, and behaviors associated with the COVID-19, psychopathic traits, and anxiety. The survey questions are displayed in the Supplementary Material section (Supplementary Table S1).

#### COVID-19 Survey

We measured both risk perceptions on the COVID-19 pandemic and the reported frequency of protective behaviors (see Pasion et al., 2020). The main variables of interest to assess risk perceptions were: (a) *COVID-19 Reaction – c*lassification of the reactions of the government to the COVID-19 pandemic using a scale ranging from 1 = too extreme to 5 = very insufficient; (b) *Penalties –* classification of penalties from 0 to 10,000€ for not following important practices to mitigate dissemination risks (e.g., to go out with COVID-19 active symptoms); (c) *COVID-19 spread –* ratio of the estimates on the number of persons who will be contaminated with the coronavirus relative to the flu; (d) *Risk of infecting others* – probability of infecting someone in the future using a 0 to 100 scale, with 0 corresponding to “not likely at all” and 100 “to very likely”; (e) *Risk of becoming infected* – probability becoming infected in the future using a 0 to 100 scale, with 0 corresponding to “not likely at all” and 100 “to very likely”; (f) *Risk perceptions on high and low risk scenarios –* based on the local health department and WHO recommendations (e.g., to receive visits vs. to receive supplies at the door) assessed from 0 – “not risky at all” to 100 – “very risky”; (g) *COVID-19 State-Anxiety* – measure adapted from the Hospital Anxiety and Depression Scale (Zigmond and Snaith, 1983; Portuguese version by Pais-Ribeiro et al., 2007) assessing anxiety states specifically related to COVID-19 circumstances (Pasion et al., 2020; e.g., “I feel tense or ‘wound up’ under the actual COVID-19 circumstances”) on a 4-point Likert scale ranging from 1 (never) to 4 (almost always). Finally, the (h) *Protective Behaviors* – frequency of engaging in protective behaviors in the last 5 days was analyzed considering the most reported official recommendations of the local health department at the time of data collection (e.g., to not physically compliment someone, to not attend to social events, and to cover the nose and the mount when coughing or sneezing) were assessed on a 100-point scale (0 – “*never”* to 100 – “*almost always”*). Composite measures of risk perceptions and anxiety toward the COVID-19 revealed satisfactory internal consistency for risk perceptions in high risk (*Cronbach’s*α = 0.78) and low risk scenarios (*Cronbach’s*α = 0.70) and good internal consistency for state anxiety (*Cronbach’s*α = 0.80).

#### Psychopathic Traits

The Triarchic Psychopathy Measure (TriPM) is a 58-item (Patrick, 2010), self-report questionnaire developed to measure the three psychopathic dimensions of the triarchic model of Psychopathy (Patrick et al., 2009): *boldness* is assessed by a 19-item subscale addressing optimism, resilience to stress, social dominance, persuasiveness, tolerance for uncertainty, self-confidence, social assurance, and intrepidness; *meanness* is assessed by a 19-item subscale addressing empathy, relational aggression, destructive aggression, physical aggression, honesty, and excitement-seeking; *disinhibition* is assessed by a 20-item subscale addressing irresponsibility, problematic impulsivity, theft, alienation, boredom proneness, impatient urgency, fraudulence, dependability, and lack of planful control. Items on all subscales are scored on a 4-point Likert scale with the following answers: false, somewhat false, somewhat true, and true. All participants completed the European Portuguese version of the TriPM (for details on the factor structure and validity see Paiva et al., 2020b). The three subscales revealed good internal consistency (all *Cronbach’s*α > 0.78).

### Statistical Analysis

First, independent block-wise multiple linear regression models were performed using the Statistical Package for the Social Sciences (SPSS, v. 25, IBM Statistics, United States) software. The models tested the combined weight of psychopathic traits on perceptions and behaviors related to the COVID-19. The models were designed with psychopathic traits (boldness, meanness, and disinhibition) as predictors and COVID-19-related variables (risk perceptions and protective behaviors) as dependent variables. All variables revealed acceptable indicators of normality with absolute values of skewness <0.99 and kurtosis <1.29. Additionally, the analysis of the scatterplots of the standardized residuals of the predictors and predicted values indicated homoscedasticity in the distribution of the residuals. Collinearity diagnosis also showed no multicollinearity among predictors (VIF < 2). Then, for those variables yielding significant associations between psychopathic traits and COVID-19 related variables, mediation models were created using the mediation model #4 of the Process plugin for SPSS (v. 3.4; Hayes, 2018) with a 10,000 samples bias-corrected and accelerated bootstrap to test the mediation role of risk perceptions in the association between psychopathic traits (predictors) and protective behaviors (outcome variable). As so, the mediation models were exploratory and designed following two main criteria: (a) the model had to include a psychopathic trait as predictor, behavior as outcome, and risk perception (e.g., perceived risk) as mediator (i.e., a model where personality traits are independent variables and risk perception is a psychological process that also influences behavior); and (b) only variables yielding significant associations in the regression analysis were included. The statistical threshold for significance was defined at α = 0.05.

## Results

Table 1 presents the Pearson correlation coefficients psychopathic traits, risk perceptions, and protective behaviors related with the COVID-19.

**TABLE 1:** Pearson correlation coefficients for age, psychopathic traits, risk perceptions and behaviors related to the COVID-19.

	**1**	**2**	**3**	**4**	**5**	**6**	**7**	**8**	**9**	**10**	**11**	**12**
1. Boldness	–											
2. Meanness	0.14	–										
3. Disinhibition	−0.10	0.63*	–									
4. COVID-19 reaction	−0.06	−0.11	−0.13	–								
5. Penalties	−0.05	−0.15*	−0.16*	0.20*	–							
6. COVID-19 spread	−0.25*	−0.09	−0.06	0.12	0.06	–						
7. Risk of infecting others	−0.10	0.06	0.08	−0.03	−0.02	−0.18	–					
8. Risk of becoming infected	−0.14*	0.10	0.10	0.01	0.02	−0.19	0.84*	–				
9. RP on high-risk scenarios	−0.10	0.02	−0.06	0.23*	0.33*	−0.06	0.22*	0.30*	–			
10. RP on low-risk scenarios	−0.06	−0.02	−0.04	0.17*	0.29*	−0.09	0.25*	0.30*	0.80*	–		
11. COVID-19 state anxiety	−0.23*	−0.02*	0.14*	0.12	0.08	0.19	0.11	0.14*	0.26*	0.31*	–	
12. Protective behaviors	−0.15*	−0.10	−0.08	0.21*	0.11	0.05	0.10	0.01	0.19*	0.11	0.13	–

*, significant at *p* < 0.05; RP, Risk Perceptions. Values in bold highlight the significant associations.

### Regression Models

Linear regression models with boldness, meanness, and disinhibition as predictors were tested for risk perceptions and protective behaviors associated with the COVID-19 pandemic. The statistics of the models and the standardized coefficients for each predictor are displayed in Table 2.

**TABLE 2:** Regression models with psychopathic traits as predictors of risk perceptions and protective behaviors.

	***Model Statistics***			***Predictors***					
		
	***F***	***AdjR*^2^**	***p***	***Boldness***		***Meanness***		***Disinhibition***	
						
				**β**	***p***	**β**	***p***	**β**	***p***
COVID-19 reaction	1.52	0.008	0.210	−0.070	0.347	−0.027	0.778	−0.122	0.205
Penalties	2.03	0.016	0.111	−0.051	0.495	−0.072	0.453	−0.114	0.232
COVID-19 spread	2.50	0.043	0.064	−**0.268**	0.002*	0.031	0.821	−0.121	0.372
Risk of infecting others	1.00	<0.001	0.393	−0.100	0.182	0.043	0.659	0.042	0.683
Risk of becoming infected	2.32	0.020	0.077	−**0.160**	0.032*	0.122	0.203	0.001	0.993
RP on high-risk scenarios	1.76	0.012	0.157	−0.132	0.078	0.151	0.117	−0.173	0.072
RP on low-risk scenarios	0.37	0.010	0.776	−0.067	0.376	0.023	0.815	−0.062	**0.523**
COVID-19 state anxiety	**5.27**	**0.062**	0.002*	−**0.194**	0.008*	−0.125	0.183	**0.203**	0.030*
Protective behaviors	2.35	0.021	0.074	−**0.168**	0.025*	−0.008	0.935	−0.097	0.310

*, significant at *p* < 0.05; RP, Risk Perceptions (dfc, dfe = 3, 190). Please note that dependent variables are displayed in rows. Values in bold highlight the significant associations.

Regarding risk perceptions, increased boldness was associated with reduced estimation of COVID-19 spread relative to flu, reduced perceived risk of becoming infected and reduced COVID-19-related state anxiety. Inversely, an increased disinhibition was significantly associated with increased COVID-19-related state anxiety. No other significant associations were found between psychopathic traits and risk perception measures. Regarding protective behaviors, boldness was the sole predictor of the frequency of protective behaviors, with increased boldness being associated with a reduced frequency of protective behaviors recommended by the local health department. No other significant associations were observed between psychopathic traits and frequency of protective behaviors.

### Mediation Models

For the mediation analysis we analyzed the mediating role of COVID-19 risk perception on the association between psychopathic traits and the frequency of protective behaviors with the constraint that only variables that yielded significant associations in the regression analysis were included. Three 10,000 samples bias-corrected and accelerated bootstrap independent mediation models were tested, each one with COVID-19 spread, risk of becoming infected, or COVID-19-related state anxiety as mediators of the association between boldness and frequency of protective behaviors recommended by the local health department.

#### Model 1: Boldness, COVID-19 Spread, and Protective Behaviors

The model exploring the role of risk perceptions in *COVID-19 spread* (mediator) on the adoption of *protective behaviors* (outcome), as explained by psychopathic *boldness* traits (predictor) indicated that: (a) *COVID-19 spread* did not predict the frequency of *protective behaviors*, β = 0.001, *p* = 0.757, and (b) there were no mediation effects as observed by the standardized relative indirect effects, 95% CI [−0.049;0.052].

#### Model 2: Boldness, Risk of Becoming Infected, and Protective Behaviors

The model exploring the role of *risk perceptions in becoming infected* by SARS-CoV-2 (mediator) on the adoption of *protective behaviors* (outcome), as explained by psychopathic *boldness* traits (predictor) indicated that: (a) the *risk of becoming infected* did not predict the frequency of *protective behaviors*, β = −0.007, *p* = 0.871, and (b) there were no mediation effects as observed by the standardized relative indirect effects, 95% CI [−0.025;0.029].

#### Model 3: Boldness, State Anxiety, and Protective Behaviors

The model exploring the role of *COVID-19-related state anxiety* (mediator) on the adoption of *protective behaviors* (outcome), as explained by psychopathic *boldness* traits (predictor) indicated that: (a) state anxiety did not predict the frequency of protective behaviors, β = 0.715, *p* = 0.178, and (b) there no significant mediation effect of anxiety as observed by the standardized relative indirect effects, 95% CI [−0.065;0.012].

## Discussion

The main goal of the present study was to provide an empirical testing of the associations between psychopathic personality traits, perceptions, and behaviors throughout the COVID-19 outbreak. The correlational analysis shows that increased boldness is associated with reduced perception of COVID-19 spread, reduced reported risk of becoming infected, reduced state anxiety, and reduced frequency of protective behaviors. Increased meanness relates with reduced estimated penalties for rule breaking, and increased disinhibition relates with both reduced estimated penalties and increased state anxiety. Importantly, when accounting for the shared variance amongst psychopathic traits, the regression analysis shows the same pattern of associations, with the exception of the associations between both meanness and disinhibition with the estimated penalties, which are not significant in the regression analysis. Additionally, the exploratory mediation analysis shows that risk perceptions related to the COVID-19 are not significant mediators of the associations between boldness and the frequency of protective behaviors.

The study of the influence of personality traits and health related behaviors is suitable for the present context of the COVID-19 pandemic. As stressed by Miguel et al. (2021), although containment measures represent an effective strategy to reduce the spread of the virus, the individuals’ adherence to such measures is influenced by personality characteristics (Carvalho et al., 2020; Miguel et al., 2021), with recent findings suggesting that antisocial and psychopathic traits predict reduced adherence to protective behaviors in response to the pandemic threat (Blagov, 2020; O’Connell et al., 2020). Besides extending the link between psychopathic personality traits and behavioral response to COVID-19 outbreak, this study adds knowledge to the available literature, once it addresses not only how these traits predict COVID-19 outbreak-related perceptions, but also how the latter mediate the association between psychopathic traits and engagement in protective behaviors. In addition, and given that risk perceptions represent a plausible explanatory mechanism for the link between psychopathic traits and patterns of risk-related behaviors (Hosker-Field et al., 2016), the present study further extends our understanding of how psychopathic traits can affect individual reactions to COVID-19 containment measures.

Stemming from the triarchic model of psychopathy, we hypothesized that boldness would be associated with reduced risk perceptions and overall reduced frequency of adopting protective behaviors (H1). Indeed, our results support this hypothesis, given that boldness was associated with reduced estimate of COVID-19 spread, perceived risk of becoming infected, state anxiety toward COVID-19, and frequency of protective behaviors. On this purpose, the well-documented relations between boldness-related traits and both lower physiological sensitivity to threat (Paiva et al., 2020a) and overweighting of reward-related behaviors (Hiatt and Newman, 2006) may provide an explanatory framework for the abovementioned association. The Risk-Return framework of Risky Choice posits that both risk perceptions and perceived benefits are strong determinants of risk-related behaviors (Blais and Weber, 2009). Individuals with high boldness may not only reveal decreased reactivity to the overall COVID-19 threat, leading to lower risk perceptions, but also an overestimation of the benefits (as opposed to risks) of engaging in risky behaviors (e.g., attend to social events, spend time with friends), hindering the engagement in protective behaviors. Nonetheless, no support was found for the mediating role of risk perceptions in the associations between boldness and the frequency of protective behaviors.

We also hypothesized that meanness would be associated with reduced estimated probability of infecting others and that both meanness and disinhibition would be related to less frequent adoption of protective behaviors (H2 and H3). However, data obtained here do not provide support for such hypotheses, since meanness was neither linked to risk perceptions, nor to the frequency of protective behaviors, and disinhibition was not associated with the frequency of protective behaviors. In fact, the sole significant association was found for disinhibition and increased state anxiety toward COVID-19 outbreak. This association highlights the possible role of state anxiety as a protective factor, favoring the adherence to protective behaviors. In fact, a previous national survey found compelling evidence for the role of anxiety in predicting the adoption of protective measures (Pasion et al., 2020). Specifically, while trait-like anxiety generally predisposes disinhibited individuals to manifest disruptive behavior by reducing the behavioral initiation threshold (Patrick et al., 2009; Paiva et al., 2020b), state anxiety related to the COVID-19 circumstances may prevent risk-taking behaviors in these individuals, as suggested by the results of the regression analysis. Finally, meanness trait entails characteristics of high interpersonal value, such as blunted empathy and lack of close attachment to others (Patrick et al., 2009), sharing trait fearlessness characteristics with boldness along with patterns of externalizing behavior with disinhibition. As we did not control neither empathic concern nor intent to place others at harm (e.g., Blagov, 2020), future studies should explore how these variables affect the association between meanness and protective behaviors, while also considering the role of empathy.

As study limitations, we would like to highlight the sample size (*N* = 194), which prevents a robust generalization to the whole population. Apart from the personality traits studied here, other features may interplay, such as prudence or consciousness, obsessive-compulsive behavior, so that further studies with bigger samples and addressing more personality traits are of extreme usefulness to better represent the whole population. Data collection was carried out during the first COVID-19 outbreak, when uncertainties regarding risks and appropriate behaviors were higher. As so, the scope of the present results is somehow limited to the first pandemic wave, but it may still add value to better understand and prevent public reactions to further COVID-19 waves or new pandemics. Finally, the present work focused on the individual responses to the health control measures imposed in by governmental action to contain the spread of the coronavirus. However, in countries, such as Brazil and even the United States, the government control measures were far less restrictive (e.g., no home confinement nor mask wearing in public spaces mandates). In the present work we did not analyzed the relation between psychopathic traits, perceptions, and behaviors in individuals with government responsibilities. By addressing this issue, future studies may contribute toward a more comprehensive analysis on how psychopathic traits may affect individual behaviors in distinct roles and contexts.

## Conclusion

As far as we know, this is the first study addressing the association between psychopathic traits, risk perceptions, and frequency of adopting protective behaviors related to COVID-19 outbreak. In the current context, both individual and collective adherence to health recommendations and spread control measures are essential to minimize the economic and social impact of COVID-19 pandemic. Our results unveil that psychopathic traits, specifically boldness, affect not only risk perceptions, but also the propensity to engage in protective measures (self and others), reducing it. By taking into account that individuals react differently to public health messages as a function of personality traits, the definition of the public health strategy and related dissemination would gain by a better adjustment to individual reactions, and possibly by implementing communication strategies specifically targeting populations with higher psychopathic traits in order to optimize engagement in protective measures.

## Data Availability Statement

The raw data supporting the conclusions of this article will be made available by the authors, without undue reservation.

## Ethics Statement

The studies involving human participants were reviewed and approved by the Local Ethics Committee, Faculty of Psychology and Education Sciences of the University of Porto. The patients/participants provided their written informed consent to participate in this study.

## Author Contributions

TP, RP, PA, and FB conceptualized and designed the study. TP and RP collected the data. TP, NC-M, and RP processed and analyzed the data and drafted the manuscript. TP, NC-M, RP, PA, and FB discussed the results and approved the final version of the manuscript. FB and PA reviewed the first draft. All authors contributed to the article and approved the submitted version.

## Conflict of Interest

The authors declare that the research was conducted in the absence of any commercial or financial relationships that could be construed as a potential conflict of interest.
