# Supplementary material for: Boldness Personality Traits Are Associated With Reduced Risk Perceptions and Adoption of Protective Behaviors During the First COVID-19 Outbreak
Source: Front Psychol. 2021 May 13;12:633555. doi: 10.3389/fpsyg.2021.633555 (PMC8155284; doi:10.3389/fpsyg.2021.633555)
Supplement: Supplementary file 1 [file Table_1.DOCX]

Table S1.

*Data sections and variables on the online Survey additional to the Triarchic Psychopathy Measure (Patrick, 2010; Portuguese version Paiva, Pasion et al., 2020a).*

| **1** | **Socio-demographic data section.**   - Sex (“*Male or Female*”) - Age (*in years*) - Educational level (“*4^th^ grade or less, 6^th^ grade, 9^th^ grade, high school, university*”) - Professional status (“*studying,* *active, unemployed, retired*”) - Workplace (“*on site or teleworking*”) - Geographic residential location (“*North”, Centre, South or Insular*”) - Rural area of residence (“*yes or no*”) - Past traumatic events (“*yes or no”*, if yes – “*war, accident, natural disaster, life-threatening diseases, physical assault from other person or an animal, sexual assault, sudden or violent death of a friend or relative, and eye witnessing a deathly situation”*) - High-risk medical conditions for COVID-19 complications according to the WHO and the Portuguese Decree Law 2-A/2020 of March defining the contingencies of the quarantine for these high-risk groups (“*yes or no”*, if yes – “*cardiovascular, oncological, and chronic diseases, hypertension, diabetes, and other*”) |
| --- | --- |
| **2** | **COVID-related data section.**   - COVID-related symptoms in the past two weeks (i.e., fever, cough, and breathing difficulties, “*yes or no”* - Known COVID-19 diagnosis in relatives or close friends (“*yes or no*”). |
| **3** | **COVID risk perceptions**.   - Risk of COVID-19 spread   Estimates on the *number of persons* who will be contaminated by COVID-19 and the seasonal flu this year in Portugal. The estimates on flu were subtracted from those of COVID-19 to isolate the risk of COVID-19 spread.   - Risk of COVID-19 contamination.   Estimates on the probability of becoming infected by COVID-19 in the future and the probability of infecting someone with COVID-19 in the future from a slider ranging from 0 to 100 (*0 corresponds to “not likely at all” and 100 “to very likely”*).   - Reactions to COVID-19 risks.   Classification of the reaction of the Portuguese Government and of the citizens using the following response scale: *1 = too extreme, 2 = somewhat extreme, 3 = adequate, 4 = somewhat insufficient, 5= very insufficient*. The mean of the two subscales was used as an index of overreactions to COVID-related risk.   - Perceived behaviour risks.   Classification of high- and low-risk scenarios based on the local health department and WHO recommendations. Each high-risk scenario was developed to have a corresponding low-risk scenario: (1) to scratch the nose after coming from the street/to scratch the nose after taking bath, (2) to receive visits/to receive supplies at the door, (3) to host a dinner party at home for friends and familiars/to telephone to friends and familiars, (4) to physically compliment someone at the street/to compliment someone at the stress with more than one meter of distance, (5) to go out to meet friends/to go out to practice exercise, (6) to not wash the hands after coming from the street/to not watch hands before waking up, and (7) to use objects that belong to other people/to use personal objects. Participants were asked to move the slider in *0 to 100 scale ranging from “not risky at all” to “very risky”*. The ratings for each scenario were used to obtain an average of High- and Low-Risk scenarios.   - Penalties.   Estimates of penalties for those not following some important practices to mitigate the risks associated with the COVID-19 dissemination: (1) to go out with COVID-19 active symptoms, (2) do not cover the nose and the mount when someone coughs or sneezes, (3) to host a dinner party at home for friends and familiars, (4) to not close a store of non-essential supplies, (5) to impede someone from working from home when it is possible, (6) to call to the local urgent health telephonic line to ask how the COVID situation is evolving. The monetary values of the penalties were presented in a slider ranged from *0€ to 10.000€.* A mean ratio of all scenarios was calculated. |
| **4** | **COVID-19 protective behaviours**   - Protective behaviours were computed considering the behavioural restrictions imposed during the quarantine by the Portuguese Decree Law, 2-A/2020 of March 20 and recommendations from the local health department. Allowed, but discouraged behaviours, during the self-mandatory quarantine in Portugal encompassed: (1) to buy food and essential supplies, (2) to go to the bank or to the post office, (3) to do exercise on the street (no more than two people together), (4) to go to the pharmacy. The most cited health recommendations were: (1) to stay at home and (2) to avoid the normal routine, (3) to not physically compliment someone, (4) to wash the hands, (5) to disinfect the surfaces, (6) to not attend to social events, and (7) to cover the nose and the mount when coughing or sneezing. At this point, it should be noticed that the Portuguese Health Department did not officially recommended the wearing of masks. The slider for assessing protective behaviours asked participants to measure the frequency of each behaviour in the last 5 days by using a scale ranging from 0 (*never*) to 100 (*almost always*). A total mean score was calculated, together with individual mean scores for allowed but discouraged behaviours during the quarantine and for the most systematic practices recommended by the local health authority. |
